# Comparative metabolomics of root-tips reveals distinct metabolic pathways conferring drought tolerance in contrasting genotypes of rice

**DOI:** 10.1186/s12864-023-09246-z

**Published:** 2023-03-27

**Authors:** Zahra Ghorbanzadeh, Rasmieh Hamid, Feba Jacob, Mehrshad Zeinalabedini, Ghasem Hosseini Salekdeh, Mohammad Reza Ghaffari

**Affiliations:** 1 Department of Systems Biology, Agricultural Biotechnology Research Institute of Iran (ABRII), Agricultural Research Education and Extension Organization (AREEO), Karaj, Iran; 2 Department of Plant Breeding, Cotton Research Institute of Iran (CRII), Agricultural Research, Education and Extension Organization (AREEO), Gorgan, Iran; 3 Centre for Plant Biotechnology and Molecular Biology, Kerala Agricultural University, Thrissur, India; 4 Department of Molecular Sciences, Macquarie University, North Ryde, NSW Australia

**Keywords:** Root Tips; Rice, Drought Stress; Root Architecture, Metabolites

## Abstract

**Background:**

The mechanisms underlying rice root responses to drought during the early developmental stages are yet unknown.

**Results:**

This study aimed to determine metabolic differences in IR64, a shallow-rooting, drought-susceptible genotype, and Azucena, a drought-tolerant and deep-rooting genotype under drought stress. The morphological evaluation revealed that Azucena might evade water stress by increasing the lateral root system growth, the root surface area, and length to access water. At the same time, IR64 may rely mainly on cell wall thickening to tolerate stress. Furthermore, significant differences were observed in 49 metabolites in IR64 and 80 metabolites in Azucena, for which most metabolites were implicated in secondary metabolism, amino acid metabolism, nucleotide acid metabolism and sugar and sugar alcohol metabolism. Among these metabolites, a significant positive correlation was found between allantoin, galactaric acid, gluconic acid, glucose, and drought tolerance. These metabolites may serve as markers of drought tolerance in genotype screening programs. Based on corresponding biological pathways analysis of the differentially abundant metabolites (DAMs), biosynthesis of alkaloid-derivatives of the shikimate pathway, fatty acid biosynthesis, purine metabolism, TCA cycle and amino acid biosynthesis were the most statistically enriched biological pathway in Azucena in drought response. However, in IR64, the differentially abundant metabolites of starch and sucrose metabolism were the most statistically enriched biological pathways.

**Conclusion:**

Metabolic marker candidates for drought tolerance were identified in both genotypes. Thus, these markers that were experimentally determined in distinct metabolic pathways can be used for the development or selection of drought-tolerant rice genotypes.

**Supplementary Information:**

The online version contains supplementary material available at 10.1186/s12864-023-09246-z.

## Introduction

Drought is a major abiotic stress that restricts crop growth, development and yield and thus has turned into a grave threat to universal food security [1]. In addition, global climate change, particularly high temperatures and erratic rainfall patterns, combined with a growing world population, is placing tremendous stress on food security and sustainability. These challenging conditions can be overcome through breeding programs to develop drought-resistant crops [2, 3]. The combined effect of drought and other abiotic stresses can reduce potential crop production by more than 50%. According to modeling simulations, drought-affected cropping regions could quadruple by the end of the twenty-first century [4]. In response to these conditions, rice employs various adaptive methods, such as building up various osmoprotectants or solutes and changes in the direction of plant growth to avoid drought [5]. Roots are the plant’s main organs that anchor it in the soil and are required for nutrition and water absorption. Favorable responses of plants to water stress is dependent on the roots’ capacity to maintain growth (i.e., modifying the root traits such as depth, density, and root angles) and maintain/increase root hydraulic conductivity [6–8]. The ability of roots to tolerate water deficiency depends on their ability to maintain adequate carbohydrate metabolism, cell wall protein composition, osmotic potential, and metabolites involved in the oxidative stress response [9, 10]. Most research has been focused on improving features in above-ground tissues to tolerate these pressures, but roots (the ‘hidden half’ of a plant’s architecture) remain an underutilized source of crop development [11]. Root System Architecture (RSA) is critical for improving nutrient and water uptake and maintaining crop yield under optimal and drought conditions [12]. Extensive root systems can help plants overcome drought, which is influenced by growth angle, root thickness, and length [13, 14]. The meristematic zone of root tips is ideal for studying these root traits because of its high mitotic rate [15]. Lowland rice with a shallow rooting system is more susceptible to drought stress, whereas upland rice with a deep rooting system is more drought-tolerant [16]. Understanding the adaptive reactions of different root sections is critical for generating better stress-tolerant rice. The omics technologies, viz. genomics, transcriptomics, proteomics, and metabolomics, have been used to uncover metabolic pathways that could be altered to overcome the effects of drought stress [16–18]. Metabolomics is a potent tool for obtaining extensive data on metabolite profiles and metabolic network analysis [19]. Various metabolite profiling methods are used to understand the plant molecular responses to drought stress and determine the metabolites’ levels in a specific metabolite pathway or class [20–22]. The metabolic profile of drought-stressed barley and maize leaves and roots indicated a substantial buildup of metabolites belonging to glyoxylate and dicarboxylate metabolism in maize roots and isoflavonoid biosynthesis in barley roots [23–25]. Further findings revealed that drought stress tolerance is linked to a signature of metabolites of different metabolic pathways [20]. Plant drought tolerance has been thoroughly characterized at various levels, but the metabolite-mediated regulatory mechanisms involved in the RSA traits of upland rice remain a mystery. Root tips have a developmental gradient from meristematic to mature cell zones, each of which responds to water stress differently and serves a specific purpose in the formation of RSA [26, 27]. Among the different zones, the meristematic zone is the most critical zone for determining roots’ length, angle, and thickness [28]. Thus, investigating the changes in the metabolite profile of the meristematic zone could be a practical approach to identifying metabolites related to drought tolerance and could help develop drought-tolerant varieties [29]. For the current study, a metabolomics approach was employed to determine the alteration of the corresponding metabolites in the root tip (5 mm) regions of IR64, a stress susceptible, shallow-rooted lowland genotype, and Azucena, a stress-tolerant, traditional upland genotype, grown in control and drought conditions to get additional molecular insights on how drought affects metabolite production. Root-tip differentially abundant metabolites (DAMs) suggested a distinct pattern of metabolite accumulation which confers drought tolerance to rice.

## Results

### Root morphological changes induced by drought stress

To evaluate the changes in root morphology and metabolic content of the two genotypes, samples were treated with severe drought stress (30% FC). Table 1 shows that the phenotypic responses of roots of both genotypes to drought stress were significant statistically (*p*-value < 0.05). Drought stress increased the mean root length and root surface area, lateral root development, and primary and secondary root diameters in Azucena (Table 1). While slightly relaxed *P*-value of 0.05 to no statistically detectable lateral root formation, diameters of the primary and secondary roots were observed in IR64. The root length in Azucena grew from 30.1 cm to 49.18 cm after drought stress induction, whereas this change was prolonged in IR64 (25.14 cm to 29.09 cm) (Table 1). Similar results were observed for the surface area parameter; thus, the mean surface area increased from 90.77 cm^2^ to 115.87 cm^2^ in stressed Azucena, whereas it changed from 70.01 cm^2^ to 77.61 cm^2^ in IR64. The numbers of root tips were also more pronounced in Azucena than in IR64 (Table 1).

**Table 1:** Phenotypic parameters of the roots assessed for both genotypes using the SmartRoot system

Genotype	Treatment	ARA	ARW	ARH	RL	SA	N.Tip	LR%	RD	t-test
IR64	Control	41.08 ± 1.01^d^	43.09 ± 2.08^d^	60.70 ± 2.47^d^	25.14 ± 1.19^c^	70.01 ± 2.14^d^	27 ± 1.17^d^	35.09 ± 2.98^d^	0.30 ± 0.02^c^	0.017
	Drought	47.10 ± 1.12^c^	53.01 ± 1.18^b^	75.25 ± 1.61^b^	29.09 ± 1.04^b^	77.61 ± 1.10^c^	30 ± 0.09^c^	46.12 ± 3.13^b,c^	0.35 ± 0.01^b^	0.048
Azucena	Control	52.14 ± 1.26^b^	46.35 ± 1.49^c^	70.15 ± 2.29^c^	30.01 ± 2.87^b^	95.77 ± 3.07^b^	35 ± 2.11^b^	50.87 ± 4.75^b^	0.35 ± 0.04^b^	0.014
	Drought	59.49 ± 1.50^a^	56.10 ± 1.15^a^	90.25 ± 4.85^a^	49.18 ± 3.14^a^	115.87 ± 3.02^a^	48 ± 3.76^a^	89.14 ± 3.69^a^	0.46 ± 0.02^a^	0.010

*ARA* Analyzed Region Area (cm^2^), *ARW* Analyzed Region Width (cm), *ARH* Analyzed Region Height (cm), *RL* Root Length (cm), *SA* Surface Area (cm^2^), *N. Tip* number of tips, *LR* lateral root (%), *RD* root diameter (cm). In each column, the same letters do not differ at significance level *P* < 0.05 (Duncan’s multiple range test)

### Metabolic changes in roots in response to drought stress

**Fig. 1:**
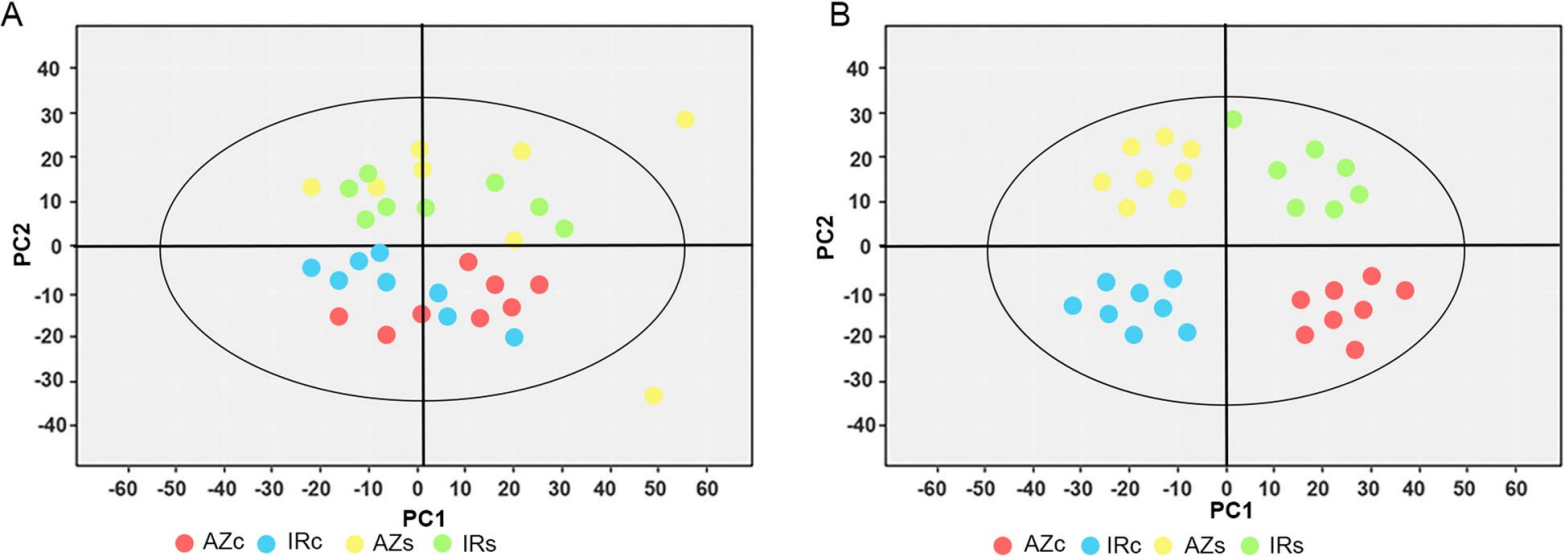
Principal component analysis (PCA) and Partial least squares-discriminate analysis (PLS-DA) score plots of metabolic profiles in rice roots under drought stress. **A** PCA score plot for Azucena normal (red), IR64 normal (blue), Azucena-treated (yellow) and IR64-treated (green) samples, **B** PLS-DA score plot for Azucena normal (red), IR64 normal (blue), Azucena-treated (yellow) and IR64-treated (green) samples

Metabolites of root tip (5 mm) regions were subjected to GC–MS to measure a wide range of metabolic changes of drought-stressed Azucena (AZs), control Azucena (AZc), drought-stressed IR64 (IRs), and control IR64 (IRc). In total, 156 metabolites were identified unambiguously and included, alkaloids, amino acids, amino acid derivatives, benzoxazinoids, fatty acid, flavonoid, lipids, nucleic acid derivatives, organic acid, phenolamides, polyphenols, sugar, sugar alcohol, vitamins and non-classified (Table S1). The metabolic data of the AZs, AZc, IRs, and IRc groups were subjected to PCA, an unsupervised multivariate data analysis method to decrease the data’s dimensionality and visualize the sample grouping. Four principal components (PCs) with explanatory and predictive values of 71.3% and 49.7% each were used to build the PCA model (Table 2). The score plot of the first two PCs is shown in Fig. 1A. The majority of the data fell inside the 92% confidence interval (Hotelling T2 ellipse). The PCA results for the four cluster samples showed that there was a clear distinction between the control (c) and treated samples (drought-stressed), while no clear difference was seen between the two genotypes (Fig. 1A). To confirm this trend, three of the whole competitive groups were examined by PCA, yielding similar results. The PCA models yielded two, two, and three PCs, respectively, for comparing AZs vs. AZc, IRs vs. IRc, and AZs vs. IRs samples, respectively. The R^2^ X and Q^2^ (goodness of prediction) values are shown in Table 2 and indicate that the differences between groups could be predictably explained by all the models. However, no distinct limit could be seen between the two groups’ PCA score plots (Table 2). PLS-DA is a supervised method that categorizes the observations into groups that yield the largest predicted indicator variable. The obtained data resulted in two PCs (R^2^ X = 0.586, R^2^ Y = 0.512, Q^2^ = 0.471) between the four cluster, and enhanced the classification between these groups in the score plot (Fig. 1B). It led to better modeling and prediction results with two PCs, when the data were examined with only the control and the treated-AZ or treated-IR64 (R^2^ Y > 0.9, Q^2^ > 0.7) (Table 2) As shown in Table 2, the treated AZ and IR samples could be separated with two PCs in spite of the slight overlap in the PLS-DA score plot (R2 Y > 0.5, Q2 > 0.08), indicating intrinsic metabolic differences between these two genotypes in the treated conditions.

**Table 2:** Explanation and predictability values of the principal component analysis (PCA) and partial least squares-discriminate analysis (PLS-DA)

		Control-stress (Azu & IR)	Control-stress (Azu)	Control-stress (IR)	Stress (Azu & IR)
PCA	R^2^X	0.713	0.742	0.759	0.659
	Q^2^	0.497	0.4015	0.462	0.4055
PLS-DA	R^2^X	0.586	0.485	0.672	0.501
	R^2^Y	0.512	0.901	0.906	0.588
	Q^2^	0.471	0.717	0.794	0.086

### Overview of Azucena and IR64 root-tips metabolome under control and drought stress conditions

**Fig. 2:**
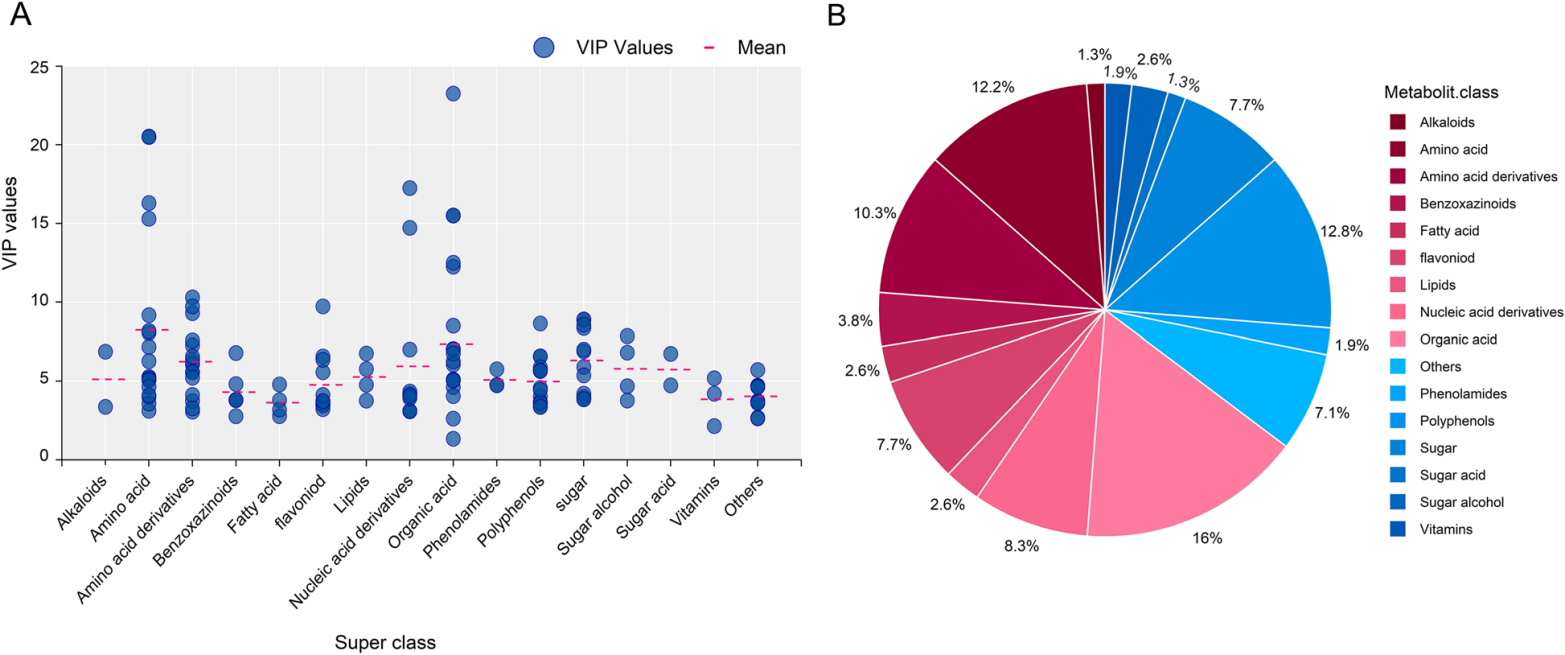
Classification of the differentially abundant metabolites and their variable importance in the projection (VIP) distribution in IR64 and Azucena in root tips. **A** VIP distribution in each metabolite superclass as a scatter plot. The average mean of the differentially abundant metabolites is shown in the red dashed line (**B**) A pie chart depicting the proportion of each metabolite in the superclass

The line plots of the X-loadings of the first component of the PLS-DA pairwise comparison models were used to identify the primary altered metabolites. Based on the parameter VIP > 1, *p*-value ≤ 0.01, log fold change (log FC > 2.0), and Kruskal–Wallis ANOVA, a total of 103 drought responsive metabolites with significant differences were identified (Table S2 and S3). The most significant metabolites had variable importance in the projection (VIP) values greater than 1, which was reported to explain the responses [30]. The VIP values for metabolites categorized by superclass are shown in Fig. 2A. Amino acid and organic acid groups were most abundant. For example, GABA and aspartic acid had a VIP value of 20.52 and 18.30, although the average value of the exclusive VIP was 5.63. Organic acid (16%), amino acid (12.2%), polyphenols (12.8%), nucleic acid derivatives (8.3%) and unclassified (others, 7.1%) were the most common differentially abundant metabolites (Fig. 2B).

**Fig. 3:**
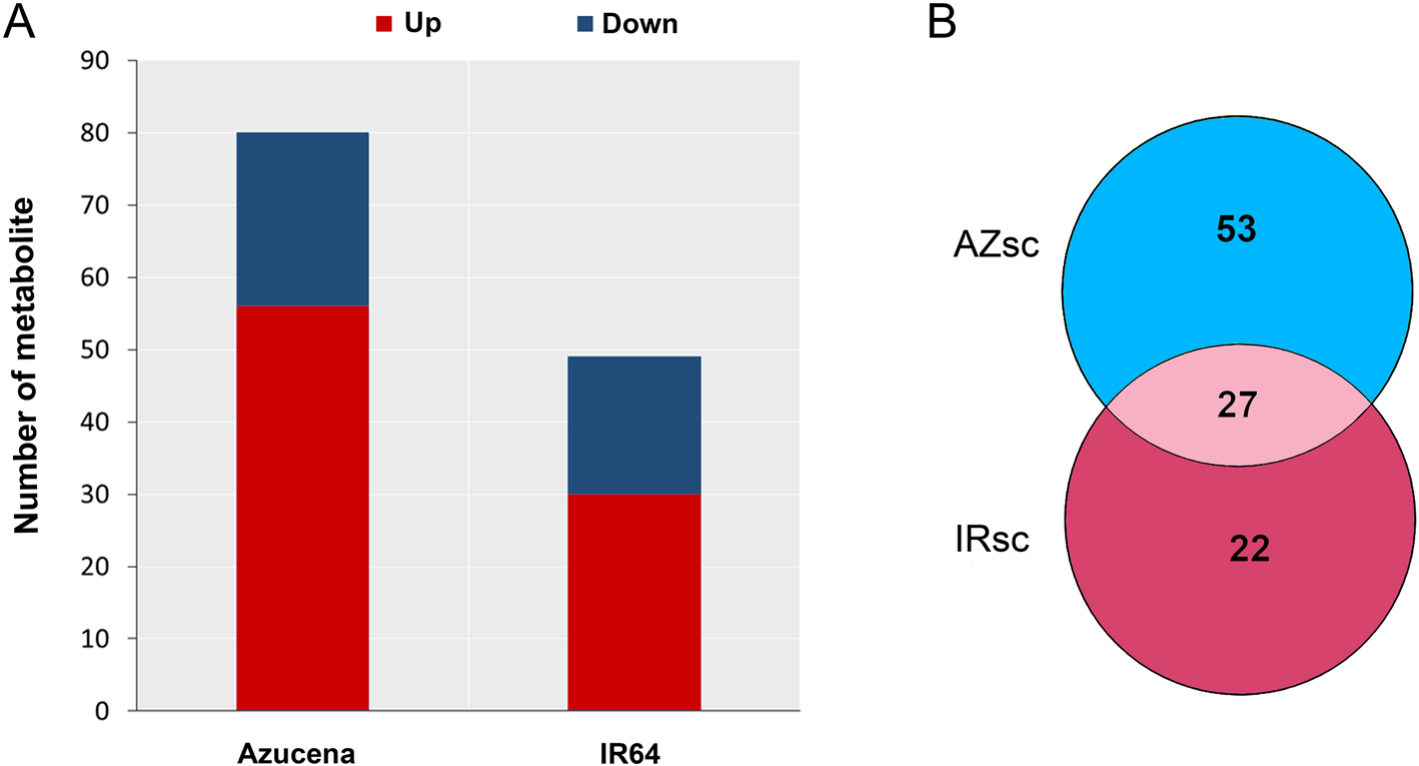
Overview of differentially abundant metabolites (DAMs) between the Azucena and IR64. **A** Up- and down-regulated metabolites of Azucena and IR64 in response to drought stress. **B** Venn diagram showing the overlap between DAMs responsive to drought stress

**Fig. 4:**
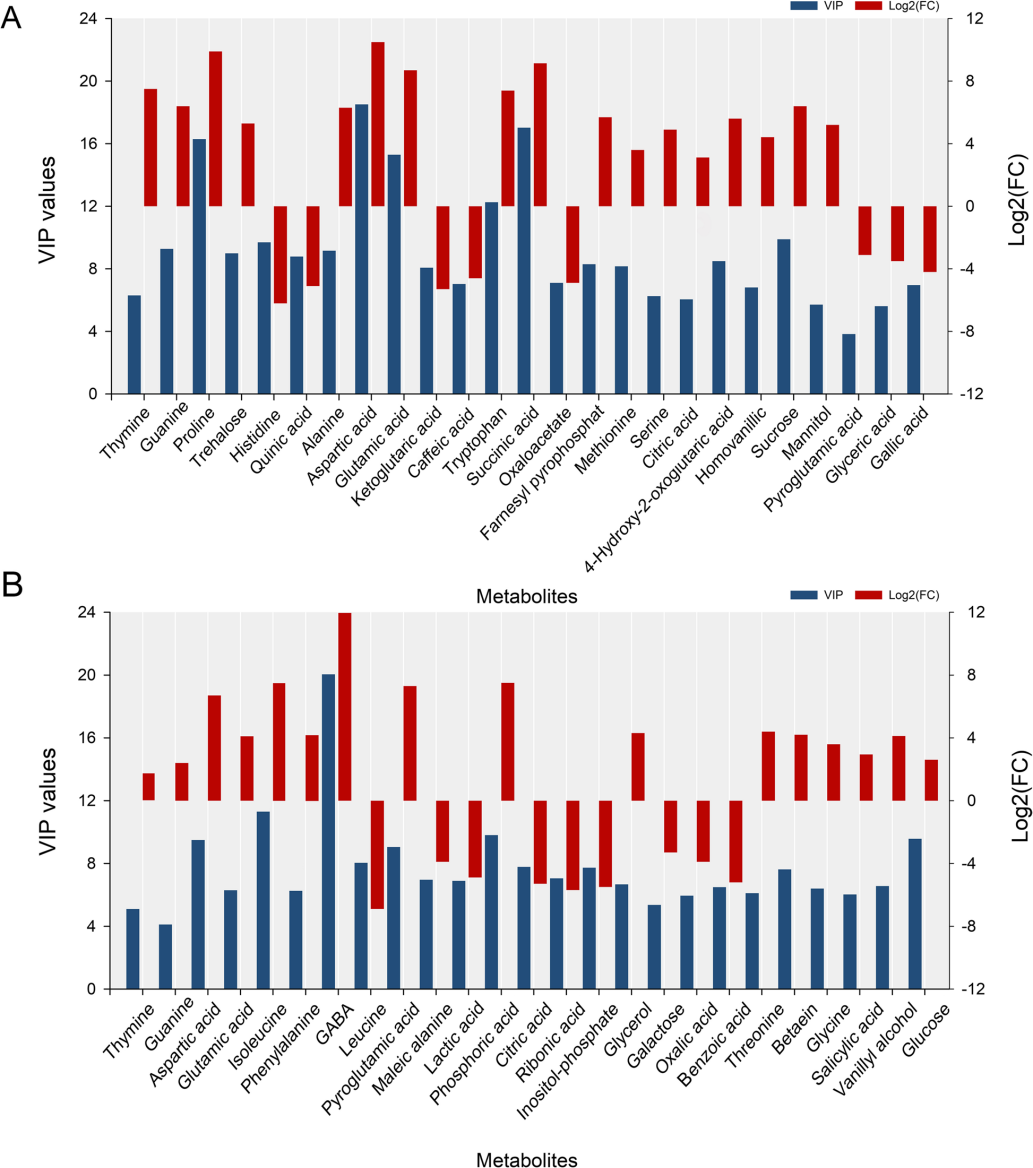
**A** Bar graph of 25 differentially abundant metabolites in Azucena root tips. VIP values are in a blue column and the red columns represent log2 (fold change, FC) values. **B** Bar graph of 25 differentially abundant metabolites in IR64 root tips. VIP values are in a blue column and the red columns represent log2 (fold change, FC) values

Compared to the control condition, the levels of 56 metabolites increased and 24 metabolites decreased in the Azucena root-tips in response to drought stress. In contrast, in IR64, the levels of 30 metabolites increased, and 19 decreased in response to drought stress (Fig. 3A). A cross-comparison of the differentially abundant metabolites between genotypes showed that number of accumulated metabolites were nearly two times higher in the root tips of the tolerant genotype (53 metabolites) than the sensitive one (22 metabolites), and of which 27 metabolites were commonly altered between the two genotypes in response to drought stress (Fig. 3B). The 25 most differentially abundant metabolites are shown in Fig. 4. The log_2_ fold change values and VIP score for these metabolites are mentioned in Table S2 & Table S3. As shown in the Fig. 4, the levels of amino acids including aspartic acid and glutamic acid, nucleotides namely thymine and guanine increased in both genotypes under drought stress. Interestingly, glycine, phenylalanine, threonine, isoleucine, and GABA were accumulated in IR64, while there was no significant difference in the levels of these metabolites in Azucena under drought stress. Likewise, there is a considerable increase in the levels of TCA cycle intermediates comprising of malic acid, isocitric acid, succinic acid and fumaric acid, and sugar and sugar alcohol such as sorbitol, mannitol, galactinol, myo-inositol, D-raffinose, sucrose, ribose and trehalose. In particular, the levels of several metabolites involved in secondary metabolism comprising of genistein, vanillin, scopoletin, conifery aldehyde, farnesyl pyrophosphate, betaine, cyanidin 3-O-rutinoside 5-O-beta-D-glucoside, and syringic acid showed the highest levels in the drought tolerant cultivar Azucena.

### Identification of potential association between metabolites and observed root traits

**Fig. 5:**
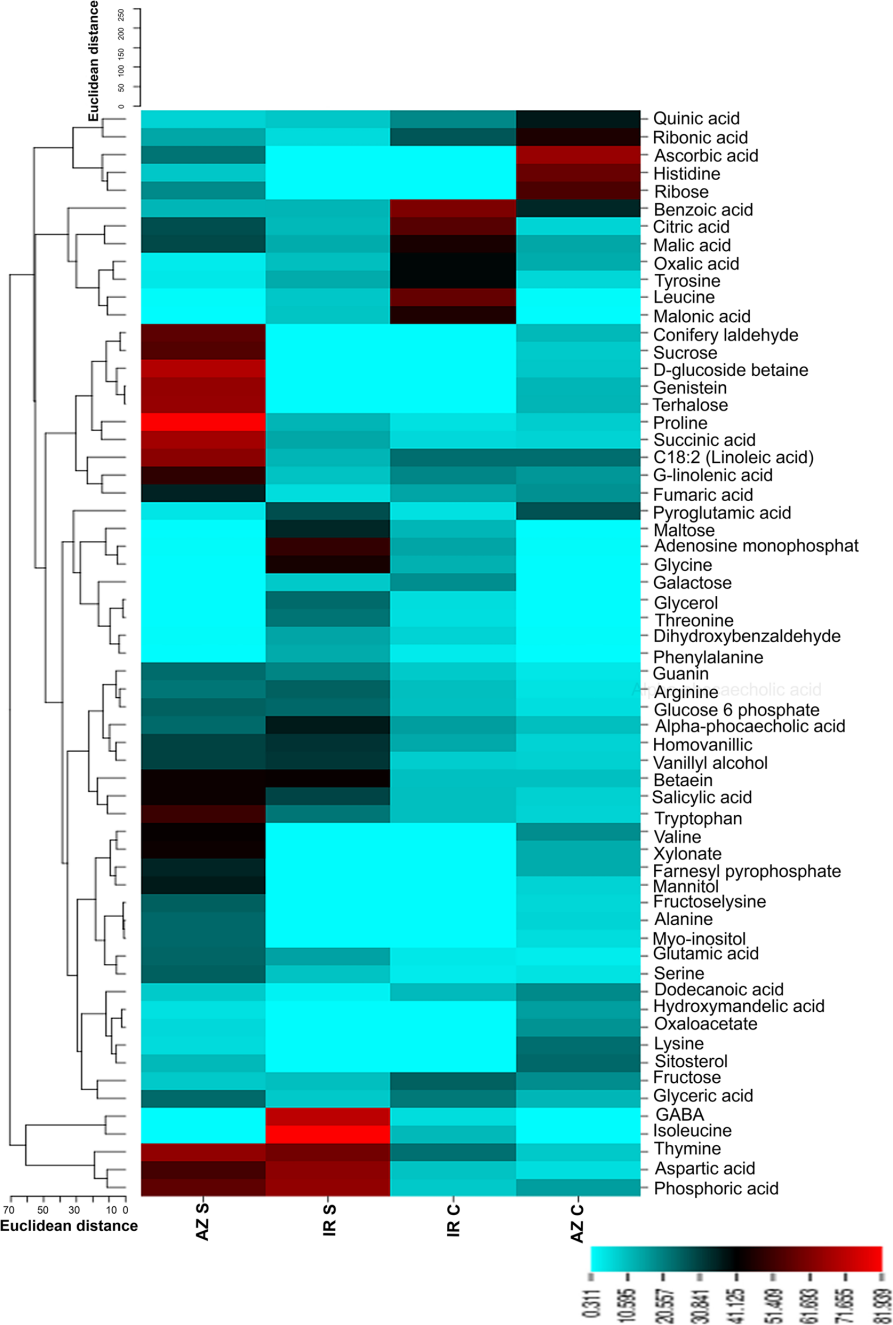
Metabolite response to drought differs between the two rice cultivars. Hierarchical clustering and heatmap of metabolite levels in root tip of IRc: IR64 genotype under control condition, IRs: IR64 genotype under drought stress conditions, AZc: Azucena genotype under control conditions, AZs: Azucena genotype under drought stress conditions

Metabolite content was determined in the root tips of control and drought-stressed plants from two different genotypes. The hierarchical clustering for both metabolites and samples (genotypes × conditions) is shown in Fig. 5. Clustering of the samples showed complete separation of the metabolite pattern between the control and drought-treated samples. Thus, drought treatment was the main source of variance in the data, indicating a complete change in metabolism under stress conditions in both genotypes. Figure 5 shows the metabolites that changed significantly under drought stress; glutamic acid, aspartic acid, proline, glucose-6-phosphate, and thymine were among the predominant metabolites that increased in response to drought stress, whereas metabolites belonging to quinic acid and ribonic acid (lowest group in Fig. 5) decreased under drought stress.

**Fig. 6:**
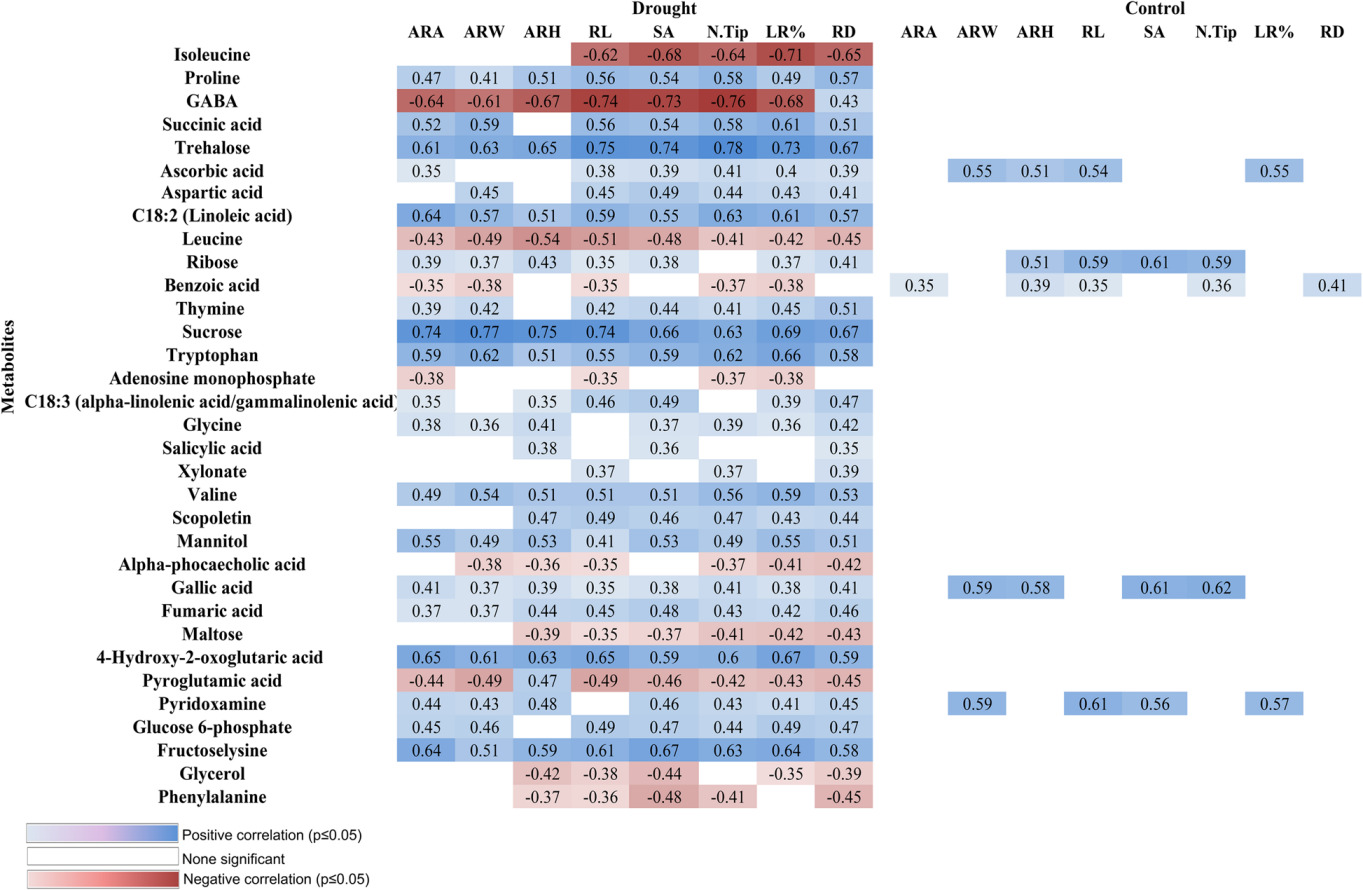
Correlation of root phenotypic data with metabolite levels. Correlation coefficients for selected metabolites with significant (*p*≤0.05) positive (blue) or negative (red) correlation between concentration metabolite levels with root phenotypic data under drought or control conditions. Data of root tip of two cultivars (Azucena and IR64) grown in two conditions. Mean values of three to five replicates per cultivar and condition were correlated. ARA (Analyzed Region Area (cm2)); ARW (Analyzed Region Width (cm)); ARH (Analyzed Region Height (cm)); RL (Root Length (cm)); SA (Surface Area (cm2)); N.Tip ( number of tips), LR ( lateral root (%)), RD (root diameter (cm)

The potential correlation between the abundant metabolites and the root phenotype were tested by analyzing the correlations of expression of the metabolite levels with phenotypic traits. We rely on the prediction marker for high concentrations since high amounts of a metabolite can be detected more accurately than low concentrations or their absence. High metabolite concentrations in tolerant cultivars are indicated by significant positive correlations of metabolites with phenotypic traits, whereas high concentrations in susceptible cultivars are indicated by significant negative correlations. In a positive correlation, the metabolite would be a tolerance marker because its higher concentrations would contribute to the tolerance. Metabolites with negative correlation are sensitivity markers. Most of the significant correlations between metabolite levels and root phenotypic parameters were found to be positive under drought stress (Fig. 6). Positive correlations were observed for the concentration of the trehalose, proline, succinic acid, tryptophan, salicylic acid, sucrose, fructose lysine and mannitol. Higher concentrations of these metabolites were associated with the number of tips, root length (cm), surface area (cm^2^), and root diameter. In tolerant plants as opposed to sensitive plants, concentrations of these metabolites were higher during drought stress.

However, for most of these metabolites, no correlations were found between concentrations under control conditions and root performance under drought. In contrast, concentrations of ribose under control conditions correlated positively with performance under drought and since there was a positive correlation between concentrations and root traits performance under control conditions also, ribose concentrations appear to be related to rate of root growth rather than drought tolerance. Gallic acid and ascorbic acid made better candidates for drought markers because their concentrations exhibited positive correlation with root performance only under drought stress conditions. Negative correlations were found for concentrations of leucine, isoleucine, pyroglutamic acid, phenylalanine, and glycerol. Higher concentrations of these metabolites were associated with decreased root length, surface area, and number of root tips. However, GABA concentration was strongly correlated with root diameter. The levels were 10 to 100 times higher in the sensitive genotype than in the tolerant genotype.

### Functional annotation of DAMs

**Fig. 7:**
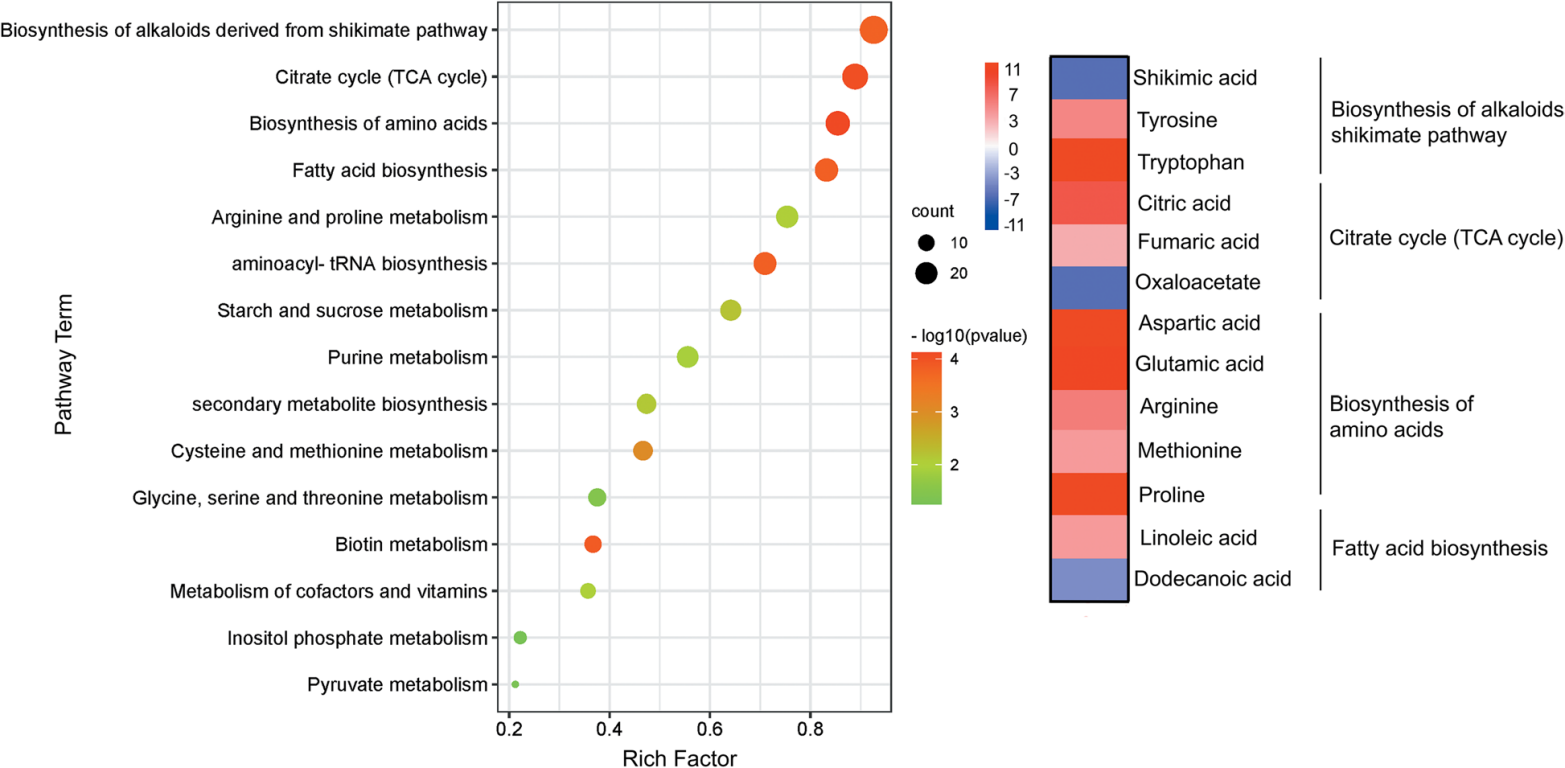
KEGG pathway classification of the differentially abundant metabolites under drought stress in Azucena (www.kegg.jp/kegg/kegg1.html)

**Fig. 8:**
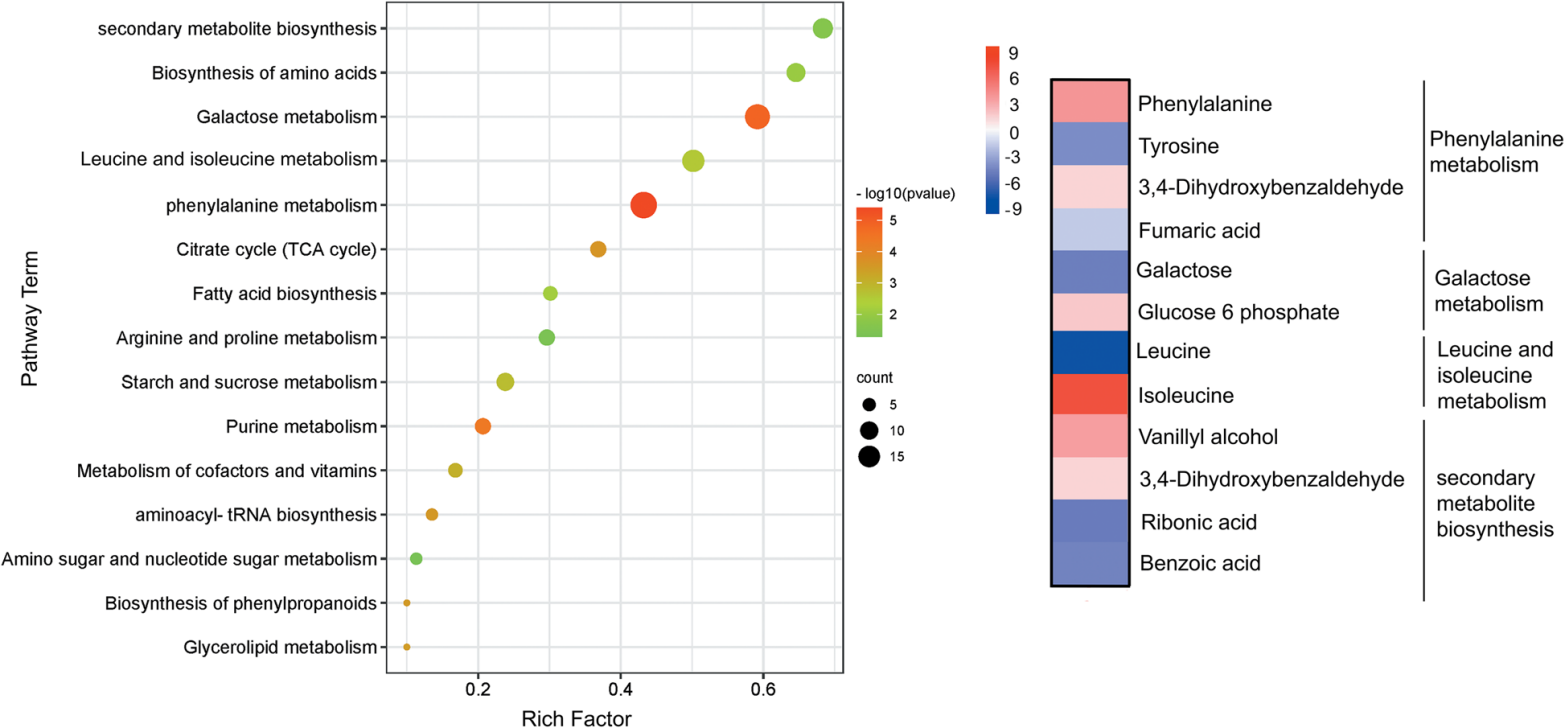
KEGG pathway classification of the differentially abundant metabolites under drought stress in IR64 (www.kegg.jp/kegg/kegg1.html)

**Fig. 9:**
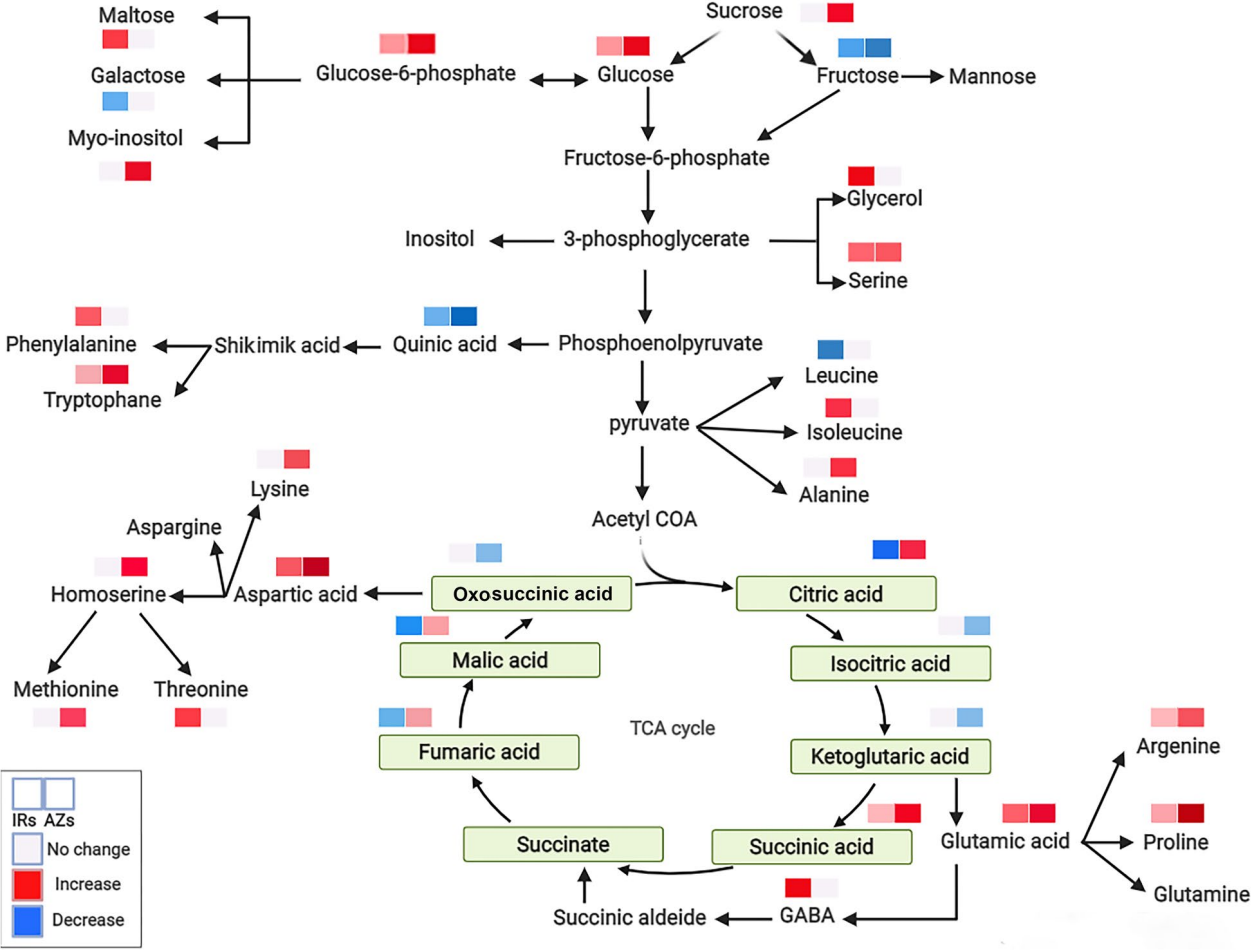
Primary metabolism responses in two rice genotypes (Azucena and IR64) after drought stress. Colors depict the relative accumulation levels of each metabolite: white (no significance), red (increase) and blue (decrease)

The differentially altered metabolites were functionally categorized based on the DAMs in Azucena and IR64, respectively, according to the KEGG database (www.kegg.jp/kegg/kegg1.html). When the control and treated plants were compared, it was observed that drought stress significantly altered the relative abundance of the levels of several metabolite classes. The most represented categories were organic acid compounds, amino acids, polyphenols, flavonoids, and sugars. Between the Azucena and IR64 genotypes, there was a significant variation in the proportion of organic acid compounds and biosynthesis of carbohydrates and amino acids. The metabolites in Azucena were mainly associated with amino acid biosynthesis and the TCA cycle, aminoacyl-tRNA biosynthesis, and fatty acid biosynthesis (Fig. 7) while metabolites in the IR64 genotype were mainly related to phenylalanine and galactose metabolism (*p* < 0.05) (Fig. 8). Increase in sucrose, glucose, tryptophan, and proline in the metabolic pathways of Azucena, may have a substantial impact on how resistant Azucena is to drought. Drought stress can have severe consequences for phenylalanine production in IR64. The high GABA expression together with its negative string correlation with root traits also indicates that IR64 restricts root elongation. Candidate DAMs demonstrating important functions or variant-specific expression profiles in Azucena and IR64 are listed in Table S2 and S3, and their relationships to major functional categories are shown in Fig. 9. A total of 103 DAMs were enriched in 30 metabolic pathways, which were most strongly represented by starch and sucrose metabolism, amino acid biosynthesis, secondary metabolite biosynthesis, purine metabolism, and fatty acid biosynthesis.

### Expression of genes involved in drought stress response

**Fig. 10:**
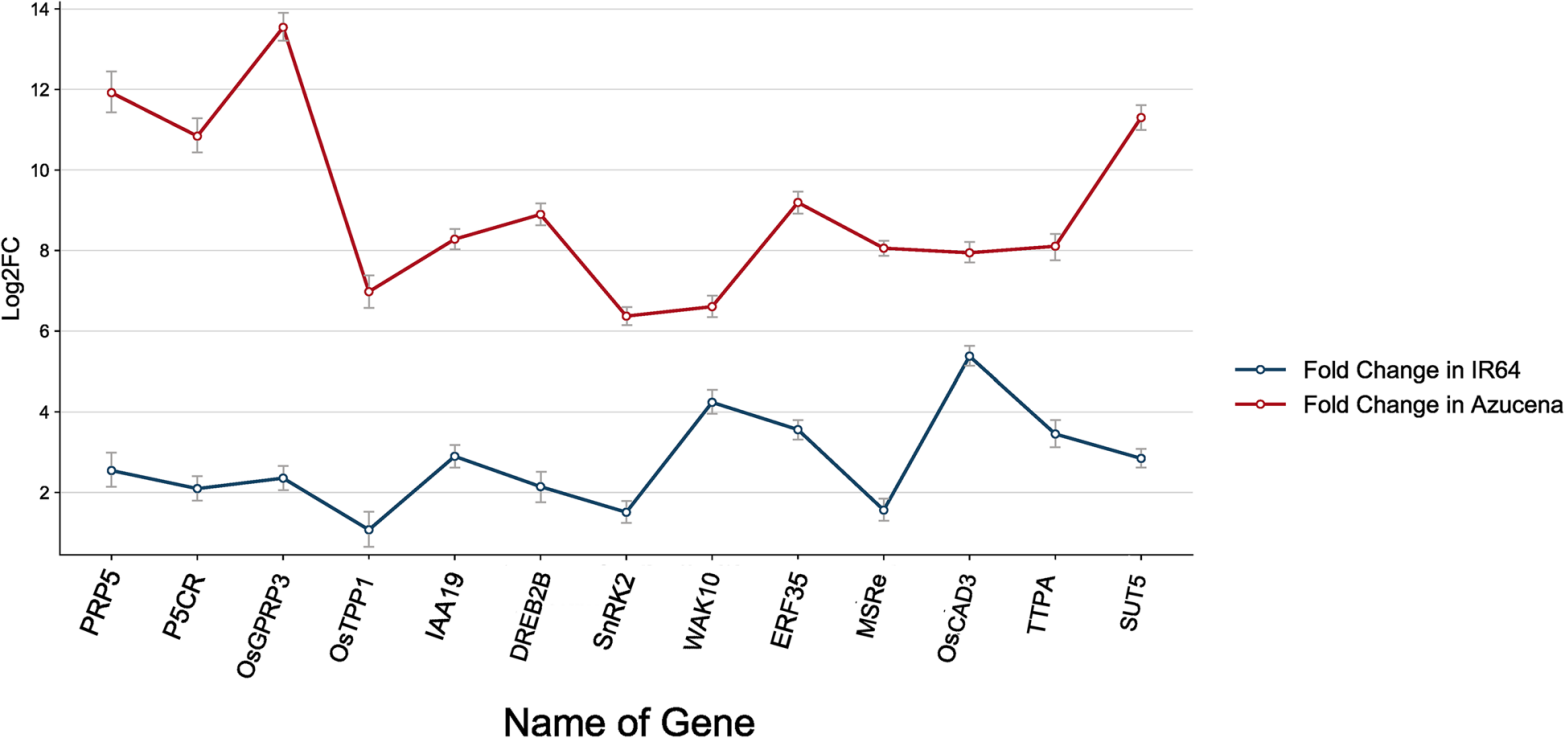
Validations of selected genes using qRT-PCR in root tip zone of both genotypes, Azucena and IR64, in response to water stress

**Fig. 11:**
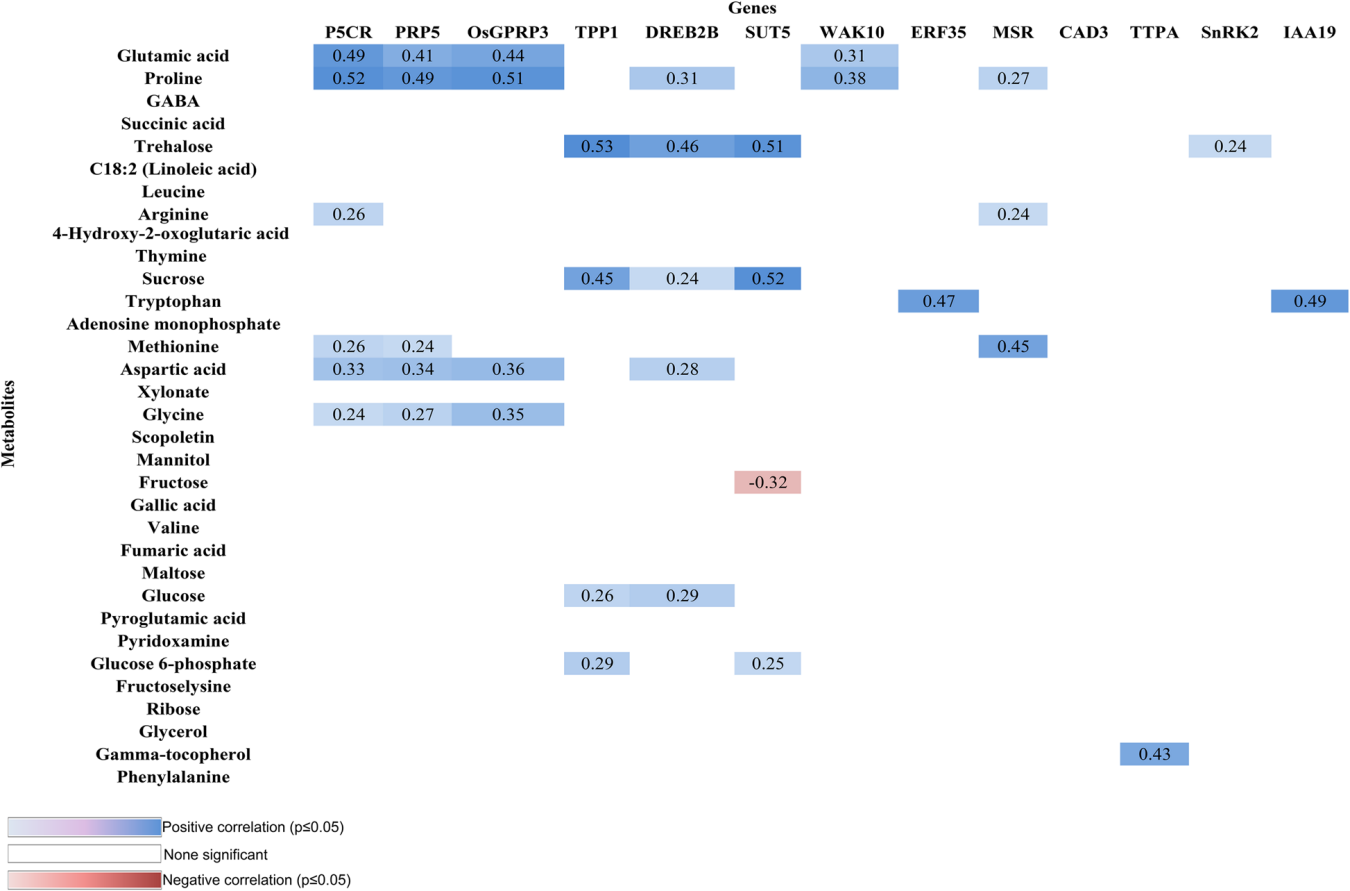
Correlation of the expression level of selected genes with the abundance of metabolites. Correlation coefficients for selected metabolites with significant (*p* ≤ 0.05) positive (blue) or negative (red) correlation between metabolite concentration and expression level of selected gene data under drought conditions

Potential marker genes involved in sugar metabolism, root growth and elongation were identified in our previous transcriptome studies on two rice genotypes with different drought tolerance [31, 32]. Thirteen genes were selected as candidate markers, and their expression levels were measured by qRT-PCR in the roots of the two genotypes grown under control and drought stress conditions. It was found that the gene Ethylene response factor (ERF35) was significantly expressed in the Azucena genotype (5.63-fold), whereas it was less expressed in the IR64 genotype (3.56-fold). Gene expression of serine/threonine kinase (SnRK2), IAA19, Trehalose -phosphate phosphate1 (OsTPP1), SUCROSE TRANSPORTER 5 (SUT5), dehydration responsive element binding protein (DREB) increased (upregulated) in Azucena by 4.86-fold, 5.38-fold, 5.90-fold, 8.45-fold and 6.75-fold, respectively, while they had no detectable change in IR64 (Fig. 10). Primer sequences and more details about genes can be found in Table S6. To find the interrelation between expression levels of selected genes and metabolites, the correlation analysis was done. The connections between metabolite levels and expression levels were in the positive rather than in the negative direction indicating a co-regulation at both the transcriptional and metabolite levels. For instance, there was a significant positive correlation between the level of proline and expression of the pyrroline-5-carboxylate reductase (P5CR) which in involved in proline metabolism (Fig. 11). Further positive connections were found between PRP5, glycine- and proline-rich protein 3 (OsGPRP3), trehalose phosphate phosphate1 (TPP1), sucrose transport protein (SUT5), and auxin-responsive protein IAA19 (IAA19) with trehalose, sucrose, and tryptophan, which were present at high levels under drought condition (Fig. 11). Thus, the high expression of these genes indicates drought tolerance under drought stress conditions.

## Discussion

### Contrasting drought tolerances of the two rice genotypes

The effectiveness of water uptake from a diverse soil environment is determined by root architecture. In drought condition due to increased soil resistance and decreased water availability, roots are unable to absorb or release water to the soil when the soil dries out. This leads to a decrease in osmotic potential and matric potential [33], as well as a decrease in turgor pressure and cell volume [34]. Root cells need to develop techniques to counteract water loss and its consequences. At times, the solution potential of the cells is reduced, increasing turgor pressure and allowing development to continue under water-deficient conditions [14, 35, 36]. The tiered structure of the root system can increase hydraulic lift and thus enhance the water uptake from deeper soil layers [37]. With the developments in breeding drought-tolerant plants, it is beneficial to focus on the characteristics of the root and patterns of root growth at different locations and time [38]. The morphological and metabolic responses of two rice genotypes with different drought stress responses were examined in this study under control and drought stress conditions. Total root growth, average root diameter, root length, and surface area were the primary morphological traits studied. Improved vertical root formation under water stress has been shown to benefit crop production [39]. Irrigation regimes have a significant effect on bread wheat root density and depth via soil water content [40]. In addition, cotton under drought stress formed thinner fine roots but longer root hairs [41]. According to these studies, under drought stress conditions, Azucena has longer roots and a large surface area for water uptake and storage. The average root length of this genotype varies from 35 to 50 cm, while the root diameter ranges from 35 to 46 μm. These values are almost double than those of IR64. These differences indicate that the two genotypes have different drought resistance mechanisms. Although root length and surface area have an effect on soil resource uptake [42], the most important sites for water uptake are the immature root tips [43]. The uninterrupted growth of root tips may be necessary for water and nutrient uptake [44]. In this study, different metabolites were detected in the two genotypes under drought stress. On the basis of the PLSDA analysis, a clear distinction was found between the metabolite content of different rice genotypes under two different water conditions. In addition to the metabolites that showed similar patterns of increase or decrease in both the rice genotypes, a few metabolites showed differences among the two genotypes. In response to water stress, 80 and 49 metabolites were detected in Azucena and IR64, respectively. In Azucena, 56 specific metabolites were accumulated in root tips, whereas in IR64, 30 different metabolites were accumulated. These results suggest that different metabolite mechanisms are involved in rice genotypes of contrasting drought tolerance. These findings suggest that rice genotypes with varying drought tolerances are affected by various metabolic pathways.

### DAMs involved in the biosynthesis of carbohydrates and energy metabolites

Increased levels of energy and carbohydrate metabolites, including those involved in sugar metabolism, were found in the Azucena in this study. Sugar is essential for plant development and affects every stage of the life cycle of the plant. It controls growth and development of stressed plants, by interacting with the plant hormones [45]. Lower sugar accumulation can lead to weak root system in IR64 genotype, while higher concentration of sugars promotes drought tolerance and behave as a signal molecule in root growth of Azucena. Sugars (trehalose and sucrose), N-containing molecules (glycine and, betaine), and polyols were all identified as suitable solvents for plant cells [46]. According to our study, the sugar content of Azucena increased when exposed to extreme water stress. Sugars (ribose, raffinose, glucose, sucrose, xylonate, and erythronate) and pyruvate, an intermediate of glycolysis, increased in Azucena roots when water was scarce. These results are consistent with a transcriptome analysis in potato and barley after drought stress. A variety of genes involved in photosynthesis, sugar metabolism, flavonoid metabolism, transporters, and transcription factors were found to be involved in the drought tolerance response in plants subjected to drought stress [47, 48]. Sucrose accumulates in many plant tissues as a result of ecological stresses such as water, salt, and cold stress [49].

Sugar alcohols, the primary photosynthetic products, function as reserves of carbohydrate in many plants, when water is scarce [50]. Mannitol has previously been shown to promote tolerance to drought and salt, in transgenic tobacco and wheat [51, 52]. Compared with the IR64, Azucena accumulated significant amounts of sugar alcohol comprising of mannitol, sorbitol, myoinositol in its roots, which could contribute to improved development and promotion of the tolerance mechanism in Azucena, while IR64 did not accumulate mannitol in its roots. Proteomics and transcriptomics analysis of drought stress in cucumber revealed that sorbitol and mannitol are among the major players in plant adaptation to salinity and drought stress [53]. Sorbitol and mannitol penetrate the cell wall and alter the cellular response to low water potential [54]. Ogbaga et al. [55], studied the response of two sorghums (*Sorghum bicolor*) cultivars Samsorg 17 and Samsorg 40, to drought stress and found substantial up-regulation of sugars (sedoheptulose, cellobiose, fructose, galactose and lactose) and sugar alcohols (ribitol, xylitol and myoinositol) in the tolerant samsorg 17. Trehalose is another metabolite that increased in Azucena when exposed to drought stress. Trehalose is an important osmolyte for osmotic control [56]. Trehalose phosphate synthase 1 (TPS1) controls trehalose metabolism and has been demonstrated to increase drought tolerance in a number of plant species [57]. Overexpression of the trehalose -6-phosphate phosphatase OsTPP3 increases drought tolerance in rice, transgenic plants show higher root length in response to drought stress [58]. Moreover, TPSP-transgenic plants exhibit less sodium ion accumulation and less damage in roots under salinity and drought stresses, and the transgenic seedlings also exhibited better growth as evidenced by greater shoot and root length [59]. Furthermore, Deyanira et al. 2012 [60] reported that trehalose metabolism serves as a biochemical marker in rice breeding because drought increased trehalose synthesis only in drought-tolerant varieties [60].

### DAMs involved in amino acid metabolite and signal sensing and transduction

Amino acids act as significant metabolites for synthesis of protein and other cellular activities in plants; they also maintain the osmotic potential of the cell, by acting as osmolytes and also scavenge reactive oxygen species (ROS) produced in drought-stressed plants [61]. According to one study, amino acids that encourage stomatal opening can likewise undo the effects of ABA or mannitol on stomatal closure, which lowers leaf evaporation [62]. Transcriptomics and metabolomics profiling of drought-tolerant and susceptible sesame genotypes, in response to drought stress, showed that ABA, proline, arginine, lysine, aromatic and branched chain amino acids, saccharopine, 2-aminoadipate, and allantoin were higher in drought-tolerant genotype under stress condition [63]. Valine, phenylalanine, isoleucine, and tryptophan content consistently increased under drought stress. Among them, tryptophan metabolism was more increased in Azucena. L-tryptophan is a physiological precursor to auxin and is crucial for reducing the impacts of drought [64]. Drought tolerance was discovered to be positively correlated with the expression level of one of the tryptophan decarboxylase genes, which was thought to be the initial enzyme gene for melatonin production [65]. Additionally, tryptophan has only been found to increase in wheat that is drought-tolerant [66]. Moreover, as tryptophan is involved in the metabolism of glycine, serine, and threonine, it is probable that tryptophan can increase plants’ ability to withstand drought. Under conditions of water stress, Azucena accumulated greater amounts of two different amino acids, fructoselysin and L-cysteinylglycine, compared with IR64. L-cysteinylglycine is a vital metabolite for numerous activities such as plant growth, development, and response to drought stress due to its unique biological properties, which are due to the presence of Cys in the chemical reactivity of GSH (- SH) and the higher water solubility of the thiol group [22]. This L-cysteinylglycine with Cys and thiol can also be thought of as a potent antioxidant in stress responses in drought-resistant genotypes. Previous studies in Arabidopsis have shown that oxidative stress increases protein glycation [56] and both glucoselysin and fructoselysin exhibit antioxidant activity [57]. The fructoselysin of the drought-tolerant genotype could also function as an antioxidant, and is responsible for drought tolerance. Isoleucine and phenylalanine are two other amino acids found in large amounts in IR64. Aside from their role in protein production, amino acids also act as the building blocks for a number of other biological processes and as precursors for a variety of secondary metabolites. They are also crucial for antioxidant activity and ROS scavenging. A low content of isoleucine helps the plant to scavenge ROS [67]. However, in IR64, the content of these amino acids is increased, which has a negative effect on the scavenging activity of ROS, resulting in lower drought tolerance of IR64.

GABA is essential for different functions, including carbon–nitrogen balance maintenance, pH control, and energy synthesis [68], and it accumulates rapidly in stressed plants, making it a stress marker. GABA content in the roots of IR64 increased significantly during prolonged drought, whereas this metabolite did not appear in the metabolite profile of the tolerant genotype. High GABA accumulation in Arabidopsis restricts cell expansion and suppresses genes involved in cell wall synthesis [69]. The accumulation of GABA under drought stress conditions in barley might be related to the suppression of primary root growth [70]. In this study, it was observed that the accumulation of endogenous GABA not only restricted root development but also reduced or delayed the formation of adventitious roots in susceptible genotype.

Arginine can increase proline level [71], which was reported to increase plant tolerance to stresses brought on by freezing, salinity, and drought [72]. Proline was the most significantly altered metabolite among all common metabolites. Proline behaves as an osmolyte for osmotic adjustment and helps in stabilization of subcellular structures, prevention of oxidative burst and is the explanation for increased drought tolerance [73]. Proline was found in higher concentrations in the roots of Azucena under stress conditions than in IR64 (39% more). Many plants have been shown to be drought-resistant when proline accumulates, and this accumulation is significantly larger in tolerant genotypes than in sensitive genotypes [7]. It was previously found that higher concentrations of proline in roots stimulate the synthesis of root hairs and root biomass, resulting in vigorous plant growth despite poor environmental conditions [16]. Due to its strong proline accumulation, Azucena root can produce more root biomass than IR64 under dry conditions. Proline has several advantages: It acts as an osmolyte, contributes to the stability of cell structure, helps scavenge free radicals and protect the redox potential of cells, improves cytoplasmic acidosis, and maintains an optimal NADP + or NADPH ratio in metabolism [7]. The greater accumulation of sugars and amino acids in the roots of the Azucena genotype may have promoted the absorption of micro- and macro nutrients, resulting in improved growth of root and thus more biomass. The lower growth of genotype IR64 during drought could be due to the synthesis of metabolites such as pyroglutamic acid that were less responsive to drought, and were produced in the roots only under stress. These findings are congruent with previous studies that have linked amino acids to plant root growth, symbiotic partnerships, and diseases in the rhizosphere [74].

### DAMs involved in TCA cycle and secondary metabolite biosynthesis

It is well understood that pyrimidine and purine nucleotides are essential for the production of lipids, peptides, secondary metabolites and carbohydrates along with being directly involved in the synthesis of nucleic acids [62]. Therefore, biosynthesis and metabolism of nucleotides are of fundamental importance in the growth, development and plant’s response to stress. Purine degradation contributes to the protective responses to drought stress, such as the buildup of the protective cellular chemical proline and the synergistic activation of abscisic acid metabolism [22]. In the current study, Azucena accumulated significantly more thymine (a purine nucleotide) and guanine (a pyrimidine nucleotide) compared to IR64 [75]. Recent studies have shown that guanine and thymine can contribute to drought tolerance of cereal genotypes through energy production and enhancement of defense responses [76].

Drought has been shown to elevate the concentration of phenolic compounds in various plants. Plants form phenolic molecules (or polyphenols) as one of their defense mechanisms against oxidative damage caused by desiccation [77]. Some drought-resistant genotypes may form more phenolic compounds with or without drought treatment [38, 78, 79]. The total content of phenolics, anthocyanins, saposides, and flavonoids in rice leaves increased after drought treatment [80]. Earlier studies indicate that phenolic compounds have a protective effect due to their unique structure, which includes double carbon bonds, hydroxyl groups, and modifications such as prenylation, methylation, and glycosylation [80, 81]. All the eight phenolic compounds found in Azucena in this study had hydroxyl groups, double carbon bonds, or methyl groups. Consequently, the phenolic components of Azucena could behave as potent antioxidants and thus prevent the oxidative damage caused in plants by drought stress.

Drought tolerance of plant species is affected by a change in the content of various organic compounds due to environmental stress conditions. The comparative content of organic matter depends significantly on the specific metabolites under the given conditions, such as succinic acid, malic acid, and galacturonic acid, which show the largest increases of all organic acids in response to long-term drought stress. Levi et al. [82] observed that increase of various organic acids, particularly citric acid, could lend to improved drought tolerance in some cotton genotypes. Malic acid increased drought tolerance in several plant species including speargrass, cotton, and tropical grasses [83, 84]. In the root, Azucena accumulated 3-dimethylallyl-4-hydroxymandelic acid, 4-hydroxy-2-oxoglutaric acid, succinic acid, salicylic acid, and isocitric acid to a considerable extent; in contrast, IR64 accumulated only phosphoric acid in higher concentrations. Another metabolite, 4-hydroxy-2-oxoglutaric acid, increased five-fold in Azucena roots during drought stress. In another study, 4-hydroxy-2-oxoglutaric acid was not detected in N. *tabacum* leaves, but in N. *tabacum* roots, a 20-fold increase was detected in the first hours (between 1 and 2 h) and a 70-fold increase after four hours of drought stress. This indicates that N. *tabacum* stores 4-hydroxy-2-oxoglutaric acid as soon as water is not available, which is then reduced to pyruvate and glyoxylate [80]. Isocitric acid, a constituent of the TCA cycle, was deposited at considerably higher levels in the tolerant genotype roots, which may have contributed to the maintenance of a vigorous root system and assimilation of nutrients and water in the current study. The TCA cycle serves as both an energy source and a carbon scaffold for the production of various amino acids [85]. The obtainability of carbon and the energy that is required for cell division influence the development of roots [86]. TCA cycle intermediates such as citric acid, malic acid and fumaric acid have been shown to be higher in stressed roots [87].

### DAMs involved in lipids and fatty acid metabolites biosynthesis

Fatty acids are important for several biological processes, including energy production and the formation of membrane lipids in living organisms. Plant defense systems also rely on fatty acid metabolic pathways [88]. Changes in lipid profiles brought on by stress cause membrane lipid remodeling and activate plant defense mechanisms in response to biotic and abiotic stresses, such as drought [89, 90]. The amount of alpha-linolenic acid in the stressed roots of the Azucena genotype was increased when compared to the control condition. Moreover, the content of alpha-linolenic acid under well-watered conditions was significantly higher in the Azucena compared to IR64, showing a genotype-specific expression pattern. Thus, the increased metabolism of linolenic acid in drought-stressed plants is consistent with the intrinsic drought tolerance of the Azucena genotype. Our results indicate that Azucena can be employed as an important genetic resource to improve drought tolerance in other cereals. According to Gundaraniya and Ambalam [91], the buildup of saturated FAs (stearic acid) was enhanced in the leaves of a drought-tolerant peanut genotype during drought stress, while the accumulation of 8,11-octadecadienoic acid decreased in the roots of a peanut genotype, that is drought-sensitive.

### Profiling of genes involved in drought stress response

In addition to metabolite profiling, qRT-PCR was executed to validate some of the major genes involved in the root growth and elongation. Our results indicated that the expression of DREB2B and IAA19 were much higher in the drought tolerance genotype. Auxin co-receptors and transcriptional repressors known as Aux/IAA proteins are essential for auxin signaling in plants. GLS content is controlled by IAA19, IAA6, and IAA5, the auxin-responsive Aux/IAA repressors. These proteins maintain high GLS expression in drought-stressed plants through a transcriptional cascade [92]. These Aux/IAA proteins are produced by the Aux/IAA genes IAA19, IAA6, and IAA5, which are directly controlled by the drought-responsive transcription factors DREB2A and DREB2B [93]. Synthesis, transport and signal transduction of auxin are essential for root elongation and adventitious root development [94]. IAA is formed via the Trp-dependent or Trp-independent pathway. Trp biosynthesis is considered an important step in both pathways [95]. Auxin is synthesized from Tryptophan via the shikimic acid pathway [96]. It has been previously shown that an increase in auxin levels promotes shoot and root development in a mixed nitrogen environment [87]. The observed changes in the stress-dependent formation of quinic acid and shikimic acid could be a cause of root elongation.

## Conclusions

The cell and tissue structure of plants is affected by drought stress. Therefore, a multidisciplinary approach (genomics, transcriptomics, proteomics, and metabolomics) would increase our knowledge of the fundamental processes of drought tolerance in rice. GC–MS Metabolomics was applied to differentiate root tip metabolites under stress and well-watered conditions. This study showed that roots from two varying genotypes subjected to drought stress, had different mechanisms for regulation and accumulation of metabolites, which is important for understanding the overall mechanisms of drought stress tolerance. Moreover, there was significant change in 103 metabolites of the root tips during drought stress, and the concentration of most metabolites increased. To maintain osmotic balance, the concentration of various compatible solutes such as sugars and polyols was increased. Positive correlations between metabolite content and drought tolerance characteristics were found for trehalose, proline, sucrose, succinic acid, aspartic acid, tryptophan, salicylic acid and mannitol. The concentrations of these metabolites were much higher in tolerant plants than in sensitive plants. Under water-limited conditions, Azucena performed better than IR64 because the root elongation rate increased more in this genotype, which could be due to the promotion of the TCA cycle in this genotype. Under drought conditions, the biosynthetic pathways of several amino acids, such as alanine, glutamate, and aspartate, was increased in the roots of Azucena and IR64. Based on the metabolomic and phenotypic changes in the roots, such as greater swelling capacity, faster transport from source to sink, and better absorption capacity, Azucena could be a promising genotype for future drought breeding programs.

## Materials and methods

### Plant material and stress treatment

IRRI provided the seeds of two known contrasting rice genotypes (*Oryza sativa* L.), Azucena (a deep-rooted upland Japonica-type genotype), and IR64 (a shallow-rooted lowland Indica-type genotype) from the International Rice Genebank Collection. Seeds were germinated on wet filter paper after sterilization. Then, the 7-day-old, equal-sized seedlings were hydroponically grown in Yoshida solution at 22–25 °C, relative humidity of 85%, and a 16 h light/8 h dark photoperiod for two weeks [97]. On the 20th day, the seedlings were transferred into root boxes filled with 1:1:2 combinations of sand, peat, and clay. The root boxes were constructed from 5 mm thick Plexiglass sheets and then transferred to the greenhouse (with the same environmental conditions). Drought stress was imposed upon the 35-day-old well-watered plants by withholding irrigation for 14 days until field capacity reached 25%-35%. Control plants were irrigated regularly. According to Puértolas [98], field capacity was randomly determined twice daily for at least 40 root boxes during the treatment period. Using a Soil Moisture Sensor SM150, the soil moisture of the 40 root boxes was randomly tested (Delta-T Devices, UK). The collection of seeds and the complete experiment was carried out according to the national guidelines [99].

### Root morphological parameters assay

After 16 days of treatment, complete roots from three plants of each genotype were collected, washed, and dried to analyze root morphological characteristics under control and stress. The SmartRoot system (https://www.quantitative-plant.org/software/smartroot) was used to measure average root diameter, length and number of lateral roots, root length, number of tips, surface area, number of branches, and overlap sections (intersections) [100, 101]. The diameter of the primary and lateral roots was determined under an optical light microscope (Olympus BX51, 10X objective).

### Sampling and metabolite extraction

Root tip sections from about 3 plants were pooled as biological replicate, and eight independent biological replicates were taken. Freshly collected root-tip samples of Azucena and IR64 genotypes were pulverized thoroughly using liquid nitrogen and mortar and pestle, and about 100 mg of this powder was taken in a 5 ml centrifuge tube. 1400 μL of pre-chilled methanol was then added to each tube and shaken for about 30 s before 60 μL of ribitol (0.2 mg mL^−1^) was supplemented as an internal quantitative control and shaken for a further 30 s [24, 102]. The tubes were then placed in an ultrasonic apparatus for 30 min at room temperature (25 °C), after which 750 μL and 1400 μL of pre-chilled chloroform and deionized water (dH2O), respectively, were supplemented and vortexed for a minute, followed by centrifugation at 14,000 r min^−1^ (4 °C) for 10 min. One ml of the supernatant was then transferred into a new tube; vacuum dried in a concentrator and then 60 μL of Methoxyamine-pyridine (15 mg mL^−1^) solution was added, vortexed (0.5 min), and allowed to react at 37 °C for two hours. To this 60 μL of the BSTFA reagent was added and the mixture was incubated for 1.5 h at 37 °C and then centrifuged at 12,000 r min^−1^ (4 °C) for 10 min and the supernatant was placed in a vial for GC–MS analysis [103].

### Untargeted metabolomics analysis

Non-targeted metabolite profiling was carried out using gas chromatography-mass spectrometry (GC-TOF–MS), according to Ghaffari et al. 2016 [104]. The injection of 1μL of each sample was done using a split ratio of 10:1 into a GC–MS, Shimadzu QP2010Plus, Japan) equipped with a DB-17 MS capillary column (0.25 mm I.D., 30 m length, 0.25 μm film thickness; Agilent Technologies Inc) at 1.2 mL min^−1^ constant flow rate. The samples in replication were continuously injected in random order to discriminate the technical variations from biological ones, as one batch. The temperatures for the ion source, the transfer line and the injector were set to 230 °C, 280 °C, and 280 °C respectively. Helium (purity 99.99%) was used as the carrier gas, flowing at a rate of 1 mL per minute. A 70 eV electron ionization was utilized in the full scan mode (50 − 1000 Da, m/z). The mass spectra provided the result data files in CDF formats, which were then imported into the XCMS software using the R software platform (http://cran.r-project.org). XCMS could be automatically preprocessed using procedures such peak detection, data baseline filtering, raw sign al extraction, and integration [105]. Ultimately, after alignment using the statistical component for comparison, the ‘CSV’ file was acquired which included data sets like retention time, sample information, and intensity of peaks. For quality control of the data (reproducibility), the internal standard ribitol was used. Additionally, all false positive peaks, such as those brought on by column bleed, noise, the BSTFA derivatization procedure, and internal standards, were eliminated from the data set. The total peak intensity of each sample was used for data set normalization, after which each sample was separately imported into the SIMCA-P software package. (Version 11.0, http://www.umetrics.com/simca). The untargeted metabolite data was processed, and the online statistical software MetaboAnalyst 5.0 (https://www.metaboanalyst.ca/) [106] was used for statistical analysis. The peak areas of the chromatograms were considered for statistical analyses.

The search program database National Institute of Standards and Technology, Gaithersburg, MD, USA (NIST) [107], the GOLM metabolome database (GMD) [108], and the Wiley Registry of Mass Spectral Library were updated with structural information and thus allowed the discovery of new and targeted metabolites. All matched spectra were analyzed and verified with authentic standards [109]. In the studies, peaks of the metabolites with more than 70% similarity index were considered effective, while those with less than 70% were deleted from the data [110]. Each metabolic component was assigned its own lane for measurement and contained amino acids, organic acids, and sugars [111]. The peak areas were normalized to the area of a single lane of the internal standard, giving the relative reaction ratios normalized by the fresh weight of each sample (log_2_-transformed).

### Metabolomics data processing and analysis

Significant analysis of metabolites (SAM) and principal component analysis (PCA) were individually utilized to pinpoint the most significant metabolites that get altered in the different genotypes during stress [112]. To identify the metabolites that were significantly changed between different conditions (control and drought), or in different genotypes (tolerant and sensitive) (*P* value < *0*.*01*), multifactorial analysis of variance (ANOVA) was executed. Additionally, Partial Least Squares Discriminant Analysis (PLS-DA) was used in this work to optimize and identify differences between the metabolic profiles of the control and drought-stressed plants. The R2 and Q2 (goodness of prediction) values, which reflected the total explained variance and the model predictability, were collected from these studies using a default seven-fold internal cross-validation. Pathway analysis was implemented for better interpretation of the functions of the altered (significantly changed at *P* < *0*.*01*) metabolites using the MetaboAnalyst 5.0 (https://www.metaboanalyst.ca) [113], via Kyoto Encyclopedia of Genes and Genomes (KEGG) pathway database (http://www.genome.ad.jp/kegg/pathway.html) and compared with *Oryza sativa* ssp. *japonica* (Rice Annotation Project Data Base http://rapdb.dna.affrc.go.jp) pathway library [114]. The t-test (*P*-value < 0.05) was performed to assess the statistical significance of the root morphology data [113].

### Correlation analysis

Correlation analysis between metabolite levels, root phenotyping and candidate gene expression data sets of paired samples was performed using Pearson correlations (function cor. test, R) according to Ghaffari et al. 2016 [104].

### Quantitative gene expression analysis

Consistent with our previous miRNA-seq and mRNA reports [31, 32], 13 genes involved in root growth and elongation and regulation of root primary metabolism were selected. Total RNA isolation was done using the TRIzol reagent (BioBasic-BS410A, Canada) as per the manufacturer’s guidelines. RNA quality was assessed on an agarose gel (1%). RNA quantity was found using a NanoPhotometer® spectrophotometer (NP80 NanoPhotometer, IMPLEN). The cDNA was reverse transcribed from the isolated RNA using the SuperScript First-Strand System for the RT-qPCR, which was performed using Invitrogen™ Kit. PerlPrimer v.1.1.21 software was used for primer designing from the transcribed region of the rice genes (sequences obtained from the RAP-DB database). qRT-PCR was accomplished using SYBR Green Master Mix (Eurogentec, Köln, Germany) in the ABI Prism 7900HT (Applied Biosystems, Foster City, CA) with the usual thermal cycling conditions (95 °C for 10 min, 95 °C for 15 s for 40 cycles, 60 °C for 1 min). The experiment was conducted in three biological replicates and two technical replicates were used. For the purpose of examining the dissociation curves for shoulders or extra peaks, the SDS 2.2.1 software (Applied Biosystems) was utilized. The expression values were normalized using the UBQ (Ubiquitin) gene as a housekeeping gene [115]. LinRegPCR was used to calculate primer efficiency [116]. “Normalized expression of the genes of interest was calculated by dividing the average relative expression (primer efficiency P to the power of cycle number Ct) of the housekeeping genes (H1) by the relative expression of the gene of interest (GOI): (GOI): ((PH1^CtH1)/2)/PGOI^CtGOI.”

## Supplementary Information

**Additional file 1:** **Table S1. **Identification results of differently accumulated metabolites between two rice genotypes under drought. **Table S2.** List of significant metabolites (between normal and drought conditions) identified through ANOVA with their *p*-value in root tip IR64. **Table S3.** List of significant metabolites (between normal and drought conditions) identified through ANOVA with their *p*-value in root tip Azucena. **Table S4.** The KEGG pathways  of the altered metabolites exposure to drought stress in Azucena root samples. **Table S5.** The KEGG pathways of the altered metabolites exposure to drought stress in IR64  root samples. **Table S6.** List of primers with details of sequence and expression profile used for RT-qPCR validation.

## Data Availability

The original contributions presented in the study are included in the article/Supplementary Material; further inquiries can be directed to the corresponding authors.

## Abbreviations

FC: Field capacity
GC–MS: Gas chromatography-mass spectrometry
PCA: Principal component analysis
PCs: Principal components
PLSDA: Partial least-squares-discriminant analysis
VIP: Variable importance in the projection
QTL: Quantitative trait locus
RL: Root length
SA: Surface area
NIST: National Institute of Standards and Technology
GMD: GOLM metabolome database
SAM: Significant Analysis of Metabolites
cDNA: Complementary DNA
DAMs: Differentially abundant metabolite
OsTPP1: Trehalose -phosphate phosphate
SUT5: SUCROSE TRANSPORTER 5
DREB: Dehydration-responsive element binding protein
PRP5: Proline-rich protein
